# Case Study on Skin Calorimetry: Modeling Localized Muscle Heat Transfer During Exercise

**DOI:** 10.3390/bios15090567

**Published:** 2025-08-29

**Authors:** Pedro Jesús Rodríguez de Rivera, Miriam Rodríguez de Rivera, Fabiola Socorro, Manuel Rodríguez de Rivera

**Affiliations:** 1 Department of Physics, University of Las Palmas de Gran Canaria, 35001 Las Palmas de Gran Canaria, Spain; fabiola.socorro@ulpgc.es (F.S.); manuel.rguezderivera@ulpgc.es (M.R.d.R.); 2 Cardiology Service, Hospital Universitario Marqués de Valdecilla, 39008 Santander, Spain; miriam.rodriguezderivera@scsalud.es

**Keywords:** sports medicine sensors, skin heat flux, skin calorimeter, skin’s thermal resistance, direct calorimetry

## Abstract

Direct measurement of heat loss in a moving limb requires attached heat-flux sensors, which are strongly affected by convection and radiation. Skin calorimetry minimizes these effects, enabling an accurate measurement. A skin calorimeter was used to measure the heat flux in the rectus femoris (thigh) of a subject exercising for 30 min at a mechanical power of 80 W. In this work, we have developed an analytical model able to describe the thermal evolution of the rectus femoris during exercise and subsequent recovery. This model consists of a sum of two exponentials *f*(*t*) = *A_1_*(1 − *e*^−*t*/*τ*^) + *A_2_*·*t*·*e*^−*t*/*τ*^, with the novelty that the second term is a linear–exponential, which opposes the first term, and that allows the initial thermal transient characterization. The time constants are the most relevant parameters, with mean values of 5 min during exercise and 10 min during recovery (for the 4 cm^2^ sensing area). The mean exercise amplitude (*A_1_*) is 1.1 mW/W, while in post-exercise it is −0.8 mW/W. In addition, the measurement of the thermal resistance of the skin before and after exercise allowed for the estimation and analysis of the evolution of the subcutaneous internal temperature, which follows the same exponential function. The developed mathematical model defines a Transfer Function (TF)—a potential invariant that can predict the thigh’s heat flux response to any exercise protocol (for the subject analyzed). This mathematical approach may be useful for sports and clinical applications.

## 1. Introduction

Up to 25% of the energy expenditure during exercise is converted into mechanical work, while the remainder is dissipated as heat [1,2]. Heat flux evolution is key to exercise performance, as the rate of muscle temperature rise strongly influences the time to exhaustion [3,4,5]. Depending on how quickly muscles heat up, fatigue may be delayed or accelerated, directly impacting endurance and overall exercise capacity. Thus, measuring heat loss during exercise is of great interest, whether assessed locally or globally. For determining the global whole-body energy expenditure, open-circuit indirect calorimetry remains the gold standard [6,7]. Accuracy and reproducibility are its major advantages, but it provides only global values and involves bulky instrumentation. 

Local methods, such as intramuscular thermistors, near-infrared spectroscopy (NIRS), and infrared thermography (IRT), enable the monitoring of parameters at the level of individual muscles. These measurements reveal that thermal behavior differs between muscles and across tissue depths [3,8], thereby supporting multilayer heat dissipation models, often addressed using complex Finite Element Method (FEM) models [9,10,11]. In exercise research, the thigh, particularly the vastus lateralis and rectus femoris, is one of the most studied sites [8,12]. These methods have also been used to study fatigue, mechanical overload [13], pathologies [14], and performance asymmetries [15]. 

Among thermal local methods, heat-flux sensors (HFSs) are the main technology capable of directly quantifying the local rate of heat loss (Wm^−2^). HFSs also enable the indirect estimation of core temperature [16], although with limited accuracy [17]. In addition, HFS technology is the basis of the in vivo measurement of skin thermal properties—a field of medical interest. Some methods have been developed to measure the skin thermal conductance [18,19,20], its heat capacity [21,22,23], and its thermal diffusivity [20,24].

The main limitation of these local thermal instruments is their sensitivity to external perturbations. Air velocity, convection, and radiation can significantly affect the measurements [25]. For this reason, these sensors are not used in clinical practice. To address these limitations, we have been developing a skin calorimeter since 2016. Applied directly to the skin, this device is capable of measuring the heat flux, the thermal resistance, and the heat capacity of the skin, with a thermal penetration depth of up to 4 mm. This novel device is specifically designed to minimize convection and radiation effects, and it is resistant to movement and air currents. Using this calorimeter, we have performed measurements at rest [26] and for the daily monitoring of pathological skin regions [27]. More recently, we developed an upgraded version for exercise applications [28,29].

The main objective of this article is to present a new application of skin calorimetry: the mathematical modelization of localized muscle heat transfer during exercise. In essence, the core of this work is a new model for localized heat loss during exercise, which consists of a sum of two exponentials f(t) = *A_1_*(1 − e^−*t*/τ^) + *A_2_*·*t*·e^−*t*/τ^, with the novelty that the second term is a linear–exponential, which opposes the first term, and that allows for initial thermal transients characterization associated with the onset and cessation of physical activity. The model is used for both heating (exercise) and cooling (recovery) phases, but with different parameters. Several experimental studies have examined thermal evolution in specific regions of the thigh [3,30,31]. A common finding is that muscle heating and cooling are asymmetric: temperature rises rapidly during exercise and decreases more slowly during recovery, remaining elevated for at least 60 min. Therefore, the use of separate models for the heating and cooling phases is justified. 

Theoretical studies based on the Pennes bioheat equation conclude that thermal evolution can be represented as the sum of decaying simple exponential functions [32,33]. Experimental studies have shown that two exponential terms are enough to describe either heating or cooling processes [34,35,36]. Accordingly, our proposed model also uses a two-exponential form. However, it introduces a novel linear–exponential term that has not been previously reported in the literature. 

On the other hand, the new two-exponential model allows for the extraction of a Transfer Function (TF) with mechanical load as the input and localized heat flux as the output. The parameters (*A_i_*, τ) of this TF are extracted from the experimental measurements. This TF allows us to easily predict the temporal response to any exercise protocol. This mathematical approach may be useful for sports and clinical applications.

In this paper, we first describe the components of the skin calorimeter, its calorimetric model, its verification process, and the methodology used to determine the heat flux and the thermal properties of the skin. We then present experimental measurements performed under different conditions. Finally, we introduce the exponential model that captures the temporal evolution of heat flux and define a Transfer Function capable of predicting thermal behavior as a function of workload.

## 2. Materials and Methods

### 2.1. Skin Calorimeter

The skin calorimeter has been described in previous works [28]. The device consists of a measurement thermopile (ET2065F2A131211W2.25 by Laird, Bromborough, UK, 13.2 mm × 13.2 mm × 2.2 mm) placed between an aluminum measurement plate (20 mm × 20 mm × 1 mm) and an aluminum thermostat (14 mm × 14 mm × 4 mm). The thermostat temperature is controlled by a custom electrical resistor (TFCC-005-50 Teflon-insulated constantan wire, by Omega Engineering, Stamford, CT, USA, 10 Ω), and its temperature is measured by a PT-100 temperature sensor (1PT100GO1020HG, Omega Engineering, Stamford, CT, USA). The cooling system, attached to the thermostat, consists of another thermopile of the same type (ET2065F2A131211W2.25 by Laird, Bromborough, UK), an aluminum heat sink (20 mm × 20 mm × 7 mm), and a fan (MF20C05, SEPA, Dessau-Roßlau, Germany). The thermostat temperature can be programmed to maintain a constant temperature or to follow a specific profile. For this purpose, a Proportional–Integrative–Derivative (PID) control is used. The thermopiles and the thermostat are surrounded by a lateral thermal insulation to minimize convection and radiation. 

**Figure 1:**
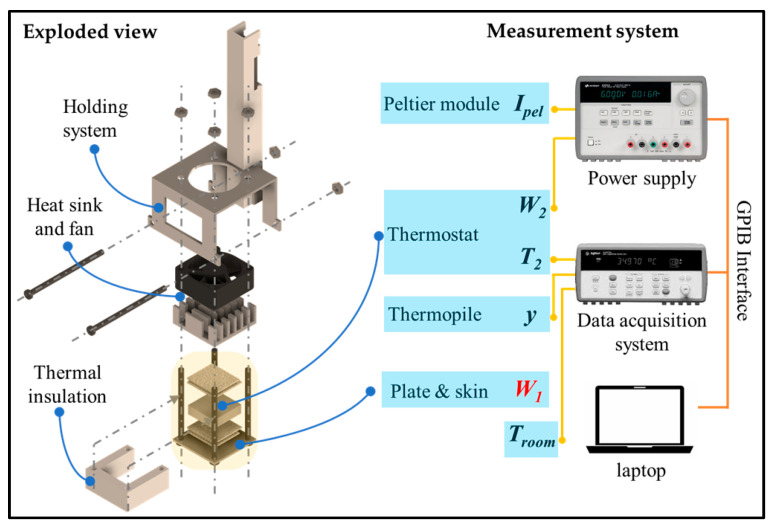
Exploded view of the skin calorimeter and measurement system scheme. The device components are indicated. The main objective of the calorimeter is to measure skin heat flux *W_1_*. For this purpose, the following signals are recorded: the calorimetric signal (*y*), the thermostat temperature (*T_2_*), the ambient temperature (*T_room_*)—measured by the Keysight 34970A and 34901A Data Acquisition System, the thermostat’s power (*W_2_*), and the Peltier current (*I_pel_*)—controlled by the Keysight E3631A triple power supply.

The main objective of the calorimeter is to measure the skin heat flux, *W_1_*. For this purpose, the following signals are recorded: the calorimetric output from the measurement thermopile (*y*), the thermostat temperature (*T_2_*), the power dissipated in the thermostat’s heating resistor (*W_2_*), the supply current of the cooling thermopile (*I_pel_*), and the ambient temperature (*T_room_*). Data are acquired using a Keysight 34970A and 34901A Data Acquisition System (Keysight technologies, Santa Rosa, CA, USA). A Keysight E3631A triple power supply powers the thermostat’s heating resistor, the cooling thermopile, and the fan. Both instruments are controlled by a laptop, via a Keysight 82357B GPIB interface using a C++ program, with a sampling period of 1 s. To maintain the calorimeter attached to the skin, a custom holding system is used. An exploded view of the calorimeter and the measurement system is shown in Figure 1.

### 2.2. Operating Model

The modelization consists of representing the calorimeter–skin system as two heat capacity elements—*C_1_* and *C_2_* (at temperatures *T_1_* and *T_2_*, respectively)—connected through the measurement thermopile, which has a thermal conductance *P_12_*. The first element represents the measurement plate and the underlying skin region affected by the heat flux *W_1_*. The second element corresponds to the thermostat, which dissipates a heating power *W_2_* due to the Joule effect. The device is thermally insulated to minimize convection and radiation effects; therefore, the model equations are based exclusively on heat conduction. Then, the power dissipated in an element (*W_i_*) equals the power stored to raise its temperature (*C_i_*·*dT_i_*/*dt*) plus the conductive heat flux to the neighboring domains (*P_i_*·(*T_i_* − *T_k_*)). Assuming that the calorimetric signal is proportional to the temperature difference, *y* = *k*(*T_1_* − *T_2_*), where *k* is the Seebeck coefficient, the following system is obtained:

(1) $$\begin{array}{l} W_{1}=\frac{C_{1}}{k}\frac{dy}{dt}+\frac{y}{k}\left(P_{1}+P_{12}\right)+C_{1}\frac{dT_{2}}{dt}+P_{1}(T_{2}-T_{01})\\ W_{2}=-P_{12}\frac{y}{k}+C_{2}\frac{dT_{2}}{dt}+P_{2}(T_{2}-T_{02}) \end{array}$$

Each element is connected to the environment through couplings of thermal conductances *P_1_* and *P_2_*. *T_01_* is the temperature of the surroundings near the calorimeter. It differs from *T_room_* due to the proximity of the human body and sensor movement during exercise. This difference, denoted as Δ*T_0_*, must be determined for each test. *T_02_* is the temperature of the cold focus. The external temperatures (*T_01_* and *T_02_*) are influenced by *T_room_* and by the supply current of the Peltier module (*I_pel_*) through the following expressions:

(2) $$T_{01}=T_{room}+\alpha I_{pel}+\Delta T_{0}\;\;\;\;\;;\;\;\;\;\;T_{02}=T_{room}+\beta I_{pel}+\Delta T_{0}$$

**Figure 2:**
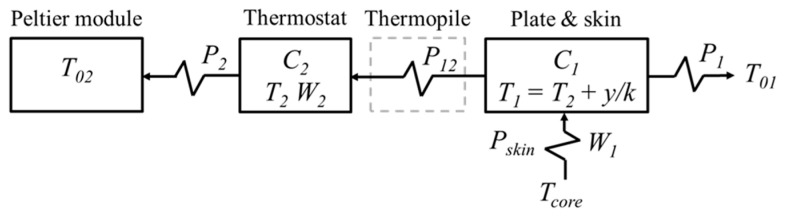
Schematic of the skin calorimeter model (Equation (1)), showing the main device components (*C_1_*, C_2_), the thermal conductances (*P_1_*, *P_2_*, *P_12_*), and temperatures associated with each element of the model (*T_1_*, *T_2_*, *T_01_*, *T_02_*).

*T_room_* is measured with a thermistor placed at a fixed location in the room. Figure 2 shows a diagram of the model, indicating the thermal conductances and the temperatures of each domain.

For calibration, specific measurements were performed to cover a wide range of the calorimeter’s operating conditions. These included varying the Peltier current (0 to 0.2 A), the Joule heating levels in the measurement plate (0 to 0.2 W), the thermostat temperature (28 to 34 °C), and the thermostat power outputs (0 to 2 W). In a steady state, thermostat temperature oscillations were ±0.02 °C and calorimetric signal oscillations were ±0.5 mV. For each measurement, the Peltier current was kept constant by setting the power supply to a higher voltage and limiting the current to the desired value.

Calibration consists of determining the model parameters by fitting both the experimental calorimetric signal and the thermostat temperature to the curves generated by the model. This fitting is performed by minimizing the Root Mean Square Error (RMSE) between curves, using the Nelder–Mead simplex method, adapted by Lagarias et al. [37], and implemented with the fminsearch function in MATLAB R2019b [38]. The calibration process begins with baseline correction to ensure that the initial and final states are steady. The parameters *C_1_*, *C_2_*, *P_1_*, *P_2_*, *P_12_*, and *k* are then determined. Once these parameters are determined, the identification process is repeated without baseline correction to calculate α and *β* (Table 1). All parameters remain invariant, except those that depend on *C_1_*, as this value is affected by the volume of skin involved in the measurement. The reported offset *C_10_* refers to the constant part of *C_1_* corresponding to the calorimeter itself.

### 2.3. Verification of the Calorimetric Model and Heat Flux Determination

To validate the model and the calibration procedure, we reconstructed the original *W_1_* signal for the 35 calibration measurements performed. In previous works, this has also been carried out under sensor movement and varying air currents [28]. The heat flux is determined as follows. First, *T_02_* is obtained from the second expression of Equation (1). Then, Δ*T_0_* is calculated from the second expression of Equation (2). Next, *T_01_* is determined from the first expression of Equation (2). Finally, *W_1_* is calculated using the first expression of Equation (1). Considering the noise of the different signals, the uncertainty of the calculated *W_1_* is 5 mW.

As an example, we present an experimental test in which both the thermostat temperature *T_2_* and the heat flux *W_1_* were varied. *W_1_* was generated by an electrical resistor embedded in a small aluminum block (10 mm × 10 mm × 4 mm) placed in direct contact with the calorimeter. This block was encapsulated in expanded polystyrene (EPS), which acted as a thermal insulator and ensured that all the heat flux was transferred to the skin calorimeter. The cooling current was maintained at *I_pel_* = 0.09 A, and the ambient temperature was *T_room_* = 21.5 °C. The thermostat temperature *T_2_* was varied from 28 to 33 °C. 

**Figure 3:**
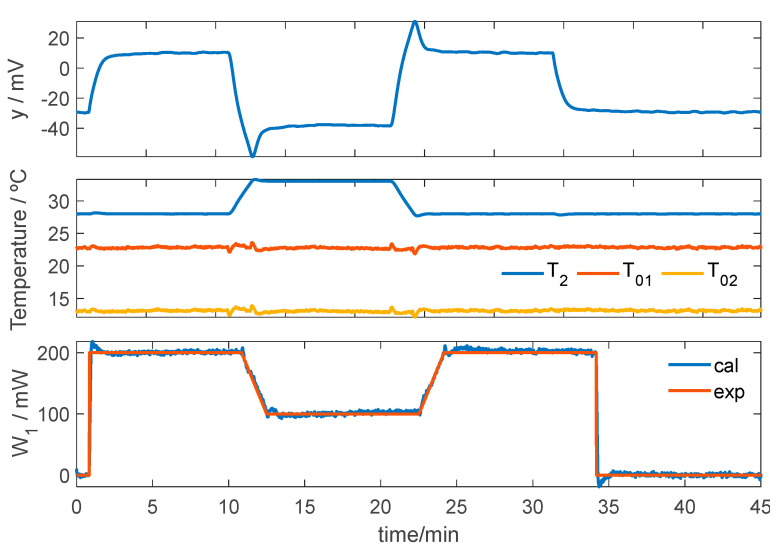
*W_1_* calculation method verification test. Calorimetric signal (*y*), thermostat temperature (*T_2_*), external temperatures (*T_01_* and *T_02_*), and experimental (*exp*) and calculated (*cal*) heat flux (*W_1_*).

Figure 3 shows the calorimetric signal (*y*), the thermostat temperature (*T_2_*), the external temperature near the calorimeter (*T_01_*), and the cold-side temperature (*T_02_*). It also displays the experimental heat flux (*exp*) and the calculated one (*cal*). These results confirm that both the modeling approach and the calibration procedure are appropriate, and that the heat flux reconstruction is satisfactory.

### 2.4. Determination of Skin Thermal Properties (At Rest)

Two thermal properties of the skin can be determined using the skin calorimeter: the heat capacity and the thermal resistance. To estimate them, a thermal excitation is applied. This excitation consists of varying the thermostat temperature (*T_2_*) while the calorimeter is in contact with the skin. The shape of the experimental signals in response to the excitation produced by the calorimeter depends directly on the heat capacity of the sample, which includes the heat capacity of the skin region being measured plus an offset value inherent to the calorimeter itself (*C_10_* value, see Table 1). By fitting the experimental response signals (calorimetric signal and thermostat temperature) to the model-reconstructed ones, using an iterative method based on the Nelder–Mead algorithm, the heat capacity *C_1_* and the heat flux *W_1_* of the skin can be determined.

On the other hand, since the subject is at rest, the internal temperature is assumed to be constant during the measurement. Under this assumption, the internal thermal resistance of the skin can be defined as the variation in skin temperature divided by the variation in skin heat flux (Equation (3)).

(3) $$R_{skin}=\frac{\Delta T_{1}}{\Delta W_{1}}=\frac{\Delta T_{2}+\frac{\Delta y}{k}}{\Delta W_{1}}$$

The obtained skin heat capacity depends on the thermal penetration depth, which depends on the duration of the temperature change [39]. In previous works, we performed several experiments to measure these thermal properties. In 2021 [26], we conducted 144 measurements in six subjects at rest, covering six zones: temple, hand, abdomen, thigh, heel, and wrist. After several studies using FEM, analytical modeling, and TF modeling, in 2025, we reported [40] that a duration of temperature change longer than 30 min is required to obtain the maximum measurable value (limited by the instrument’s uncertainty). However, a 5 min measurement reaches approximately 80% of this maximum value and is sufficiently practical for these studies [40]. In contrast, the thermal resistance depends on the steady-state values of both the calorimetric signal and the heat flux. Therefore, its accuracy relies on how well these signals stabilize. For the skin calorimeter, five minutes are enough to reach a steady state, assuming the subject remains thermally at rest.

### 2.5. Determination of Skin Heat Flux and Subcutaneous Temperature (At Exercise)

To determine the skin heat flux (*W_1_*), we use the procedure explained in Section 2.3. However, in this case, the thermostat temperature is constant. Thus, we can obtain a simplified expression of the following form for each skin calorimeter (S1 and S2):

(4) $$\begin{array}{l} W_{1}=7.0805y-2.8159I_{pel}+0.50877W_{2}\left(S1\right)\\ W_{1}=7.1708y-2.9348I_{pel}+0.52727W_{2}\left(S2\right) \end{array}$$

where *W_1_* and *W_2_* are in watts, *y* is in volts, and *I_pel_* is in amperes. The coefficients are calculated from the calibration parameters (Table 1). Section 2.3 of this paper describes the reconstruction of the heat flux in a case of Joule dissipation, validating both the model and the calculation method.

Regarding the internal subcutaneous temperature, and according to the calorimeter–skin model shown in Figure 2, its estimation requires knowledge of the skin’s thermal resistance (*R_skin_*). This parameter is calculated as the average of the values obtained from specific measurements with the subject at rest, conducted before and after exercise. The internal temperature is then computed from the estimated heat flux and the experimental curves of thermostat temperature and calorimetric signal (Equation (5)):

(5) $$T_{core}=T_{1}+W_{1}R_{skin}=T_{2}+\frac{y}{k}+W_{1}R_{skin}$$

## 3. Results and Discussion 

### 3.1. Experimental Measurements

To experimentally study heat flux during exercise, six long-duration (2 h) measurements were performed on a healthy male subject (30 years old, 90 kg, 1.80 m). The exercise protocol for all measurements consisted of 30 min of continuous activity performed on a stepper at a constant mechanical power output of 80 W. The power output was maintained by monitoring the stepping rate. 

**Figure 4:**
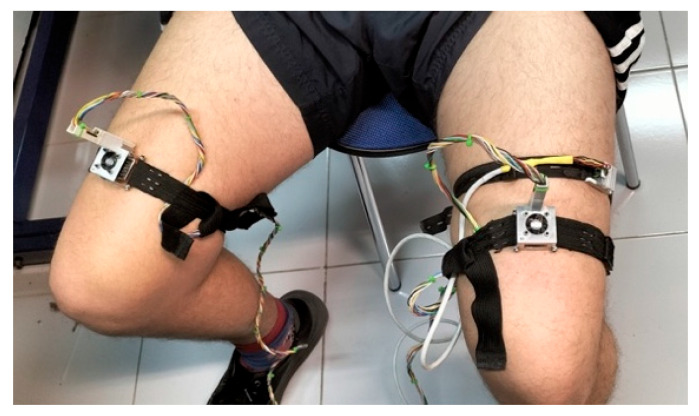
Placement of the skin calorimeters for the measurement of skin thermal properties.

**Figure 5:**
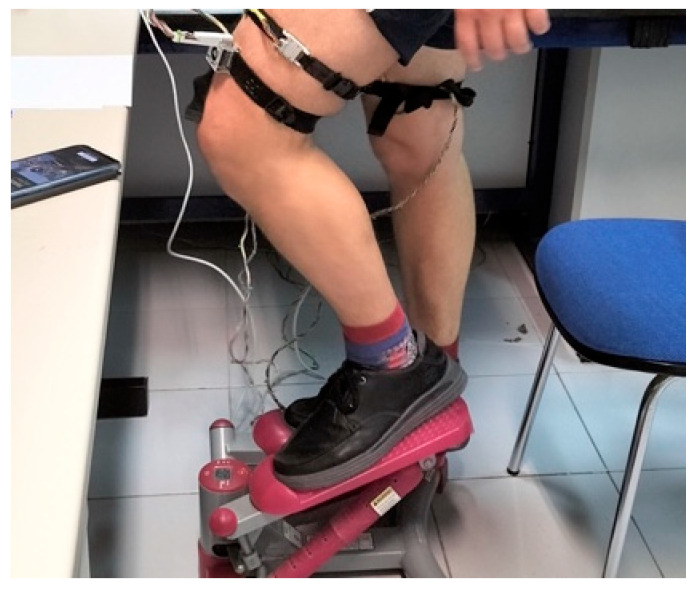
Subject performing exercise on a stepper with skin calorimeters placed on the thigh.

Heart rate was monitored throughout the session using a finger pulse oximeter, with an average initial rate of 85 bpm and a peak of 165 bpm. A skin calorimeter was placed on each thigh (rectus femoris). For each trial, the thermostat temperature was kept constant but set to a different value: 28, 30, 32, 34, 36, or 38 °C. The average ambient temperature was 22.5 °C, with a relative humidity of 45%. Before and after the exercise, additional measurements were conducted to determine the thermal properties of the skin, using a thermostat temperature step of Δ*T_2_* = 4 °C. Figure 4 and Figure 5 show the location of the skin calorimeters on the subject. 

**Figure 6:**
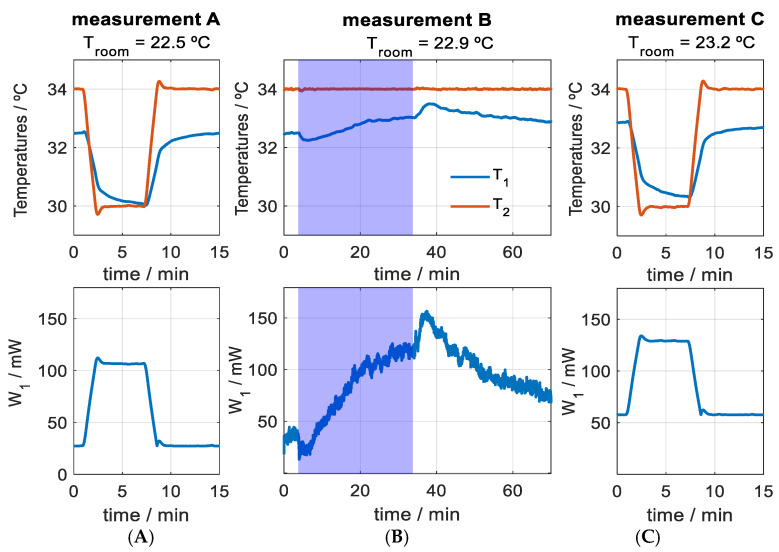
Measurement before (**A**), during (**B**), and after exercise (**C**). Tests **A** and **C** are used to determine skin thermal properties, and test **B** is used to evaluate heat flux during exercise. The blue area on measurement **B** corresponds to exercise period. Skin temperature (*T_1_*, blue), thermostat temperature (*T_2_*, red), and total skin heat loss (*W_1_*) are shown for a measurement surface of 4 cm^2^.

Figure 6 presents a complete measurement, displaying the skin temperature (*T_1_*), the thermostat temperature (*T_2_*), and the calculated heat flux (*W_1_*), using the procedure explained in Section 2.4. The pre- and post-exercise measurements were used to determine the heat capacity and the internal thermal resistance of the skin. In the case shown, the average heat capacity was *C_skin_* = 3.0 J/K (for a measurement area of 4 cm^2^), and the average thermal resistance was *R_skin_* = 30.1 K/W. The measurement uncertainty for the thigh zone, determined in a previous work [26], was 0.1 J/K for heat capacity and 1.3 K/W for thermal resistance. Since the temperature change used to determine these thermal properties was 4 °C, the reported value of *R_skin_* corresponds to the intermediate temperature.

**Figure 7:**
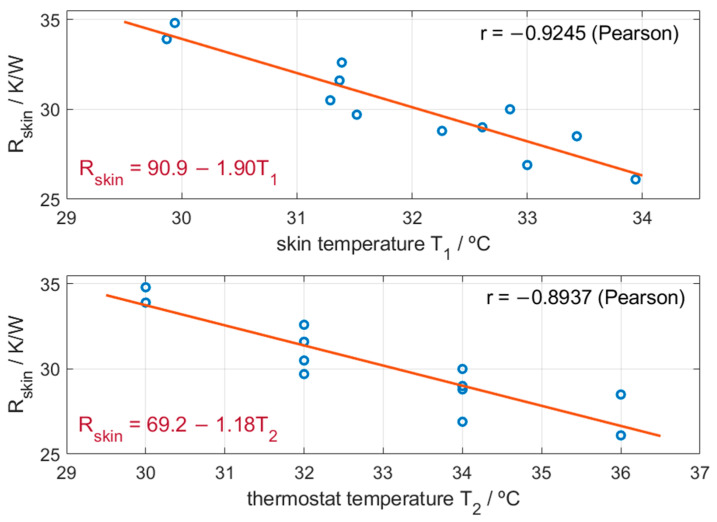
Internal skin resistance for a measurement surface of 4 cm^2^ (*R_skin_*) as a function of skin temperature (*T_1_*) and thermostat temperature (*T_2_*). Experimental values (blue circles) and linear fit (red line).

Figure 7 shows the internal thermal resistance of the skin as a function of the associated temperature. A strong Pearson correlation was found with both the skin temperature *T_1_* (*r* = −0.9245) and the thermostat temperature *T_2_* (*r* = −0.8937). This decrease in internal resistance as a function of temperature may reflect a physiological response, such as a slight thermally induced vasodilation, which could enhance skin heat transfer.

### 3.2. Mathematical Model of Heat Flux and Internal Skin Temperature Evolution

**Figure 8:**
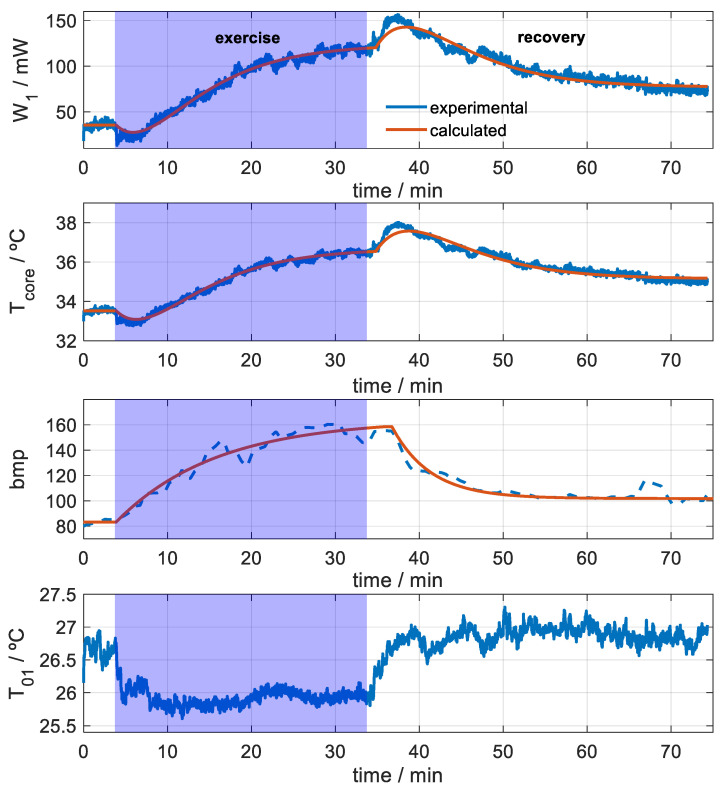
Measurement on the thigh (rectus femoris) of a subject exercising on a stepper at a constant mechanical power of 80 W. This figure shows the case in which the calorimeter thermostat was set to *T_2_* = 34 °C. The ambient temperature was 22.6 °C and the relative humidity was 50%. Skin heat loss (*W_1_*) for a measurement surface of 4 cm^2^, internal thigh temperature (*T_core_*), heart rate (bpm), and temperature around the calorimeter (*T_01_*) are shown, in blue (experimental) and red (modeled).

In previous Section 2.3 and Section 2.5, we described the method used to determine the temperature *T_01_* around the calorimeter, which is required to calculate the heat flux *W_1_*. Once the heat flux *W_1_* and the internal skin resistance have been determined with the subject at rest (Figure 7), the internal temperature is calculated using Equation (5). Figure 8 shows the signals of one of the measurements (*T_2_* = 34 °C). The heat flux transmitted by the skin (*W_1_*) for a measurement surface of 4 cm^2^, the internal temperature of the thigh (*T_core_*), the heart rate (bpm), and the temperature around the calorimeter (*T_01_*) are shown. As we can see, moving the calorimeter causes a slight decrease in *T_01_*.

In the search for a simple analytical function to fit the calculated skin heat flux and internal temperature, we opted for exponential functions, according to the Pennes bioheat equation [32,33,34,35,36]. Since the initial transients could not be fitted, we suspected the presence of an additional phenomenon not compatible with the Pennes equation, which is reasonable because blood perfusion varies during physical exercise. For this reason, a linear–exponential term was introduced. This term has not been previously reported for the human body. However, it is common in physical systems able to oppose to an applied action. The selected function, based on the fitting results, is given by Equation (6) and consists of three terms: the first is the initial steady-state value; the second corresponds to the increase (during exercise) or decrease (during post-exercise); and the third represents a transient exponential pulse, defined by a linear–exponential form.

(6) $$f(t)=A_{0}+A_{1}\left(1-e^{-t/\tau }\right)+A_{2}te^{-t/\tau }$$

Figure 8 shows the fit of the signals. Table 2 lists the parameters of this function (Equation (6)) for skin heat flux (*W_1_*), and Table 3 shows the fitting for internal temperature (*T_core_*). All the experimental measurements fit to the functional proposed by Equation (6). The fitting was carried out using the Levenberg–Marquardt algorithm, implemented in MATLAB’s *lsqcurvefit* function.

The parameter *A_0_* in Equation (6) corresponds to the initial heat flux value, when the subject is at rest with the calorimeter applied on the skin. This value depends on the thermostat temperature *T_2_* and the ambient thermal conditions, and it reflects the subject’s thermal state at rest. All tests were performed under similar ambient temperature and humidity levels. We observed that the initial heat flux value generally decreases as the thermostat temperature increases. However, the subject’s internal temperature at the time of measurement is not always the same, which can affect the heat flux, as we have confirmed experimentally.

**Figure 9:**
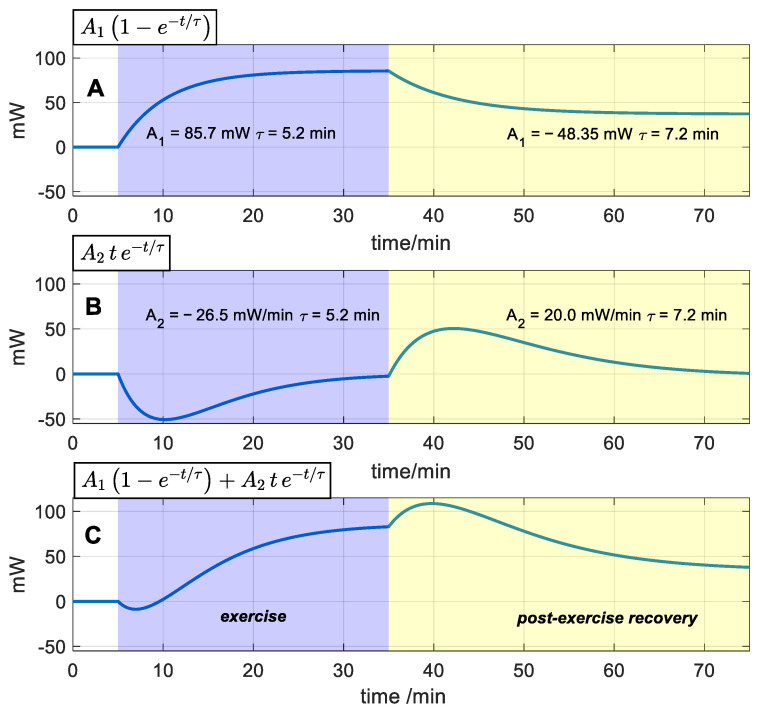
Decomposition of the heat flux increase Δ*W_1_*(t) for the case of W = 80 W and *T_2_* = 34 °C. (**A**): simple exponential term, (**B**): linear–exponential term, and (**C**): sum of (**A**,**B**). The exercise phase is shaded in blue, and the post-exercise recovery is in yellow.

As explained in Equation (6), the heat flux response is the sum of three terms. The parameter *A_0_* represents the baseline of the signal. The other two terms (with amplitudes *A_1_* and *A_2_*) are directly linked to exercise and recovery. To better understand their differences and meaning, Figure 9 illustrates the functional form associated with *A_1_* and *A_2_*.

The parameter *A_1_* corresponds to the heat flux increase due to exercise. This parameter represents the amount of heat loss through the skin by conduction due to the exercise performed (Figure 9A). In general, its value decreases slightly as the thermostat temperature increases within an intermediate range of thermostat temperatures (*T_2_* = 30–34 °C). At the extremes, this relationship becomes more pronounced, probably because excessive heating or cooling can perturb the skin’s thermal state. Our data indicate that a thermostat temperature of approximately 30–34 °C appears to be optimal under the ambient conditions of these experiments, as it provides the highest signal-to-noise ratio. A previous work suggests that *A_1_* is independent of thermostat temperature [28], but experimental measurements reveal this dependence. Therefore, to thermally study a subject, it is advisable to conduct measurements under consistent ambient and thermostat temperatures.

The amplitude *A_2_* is associated with the transients at the onset and cessation of exercise (Figure 9B). The area of the initial transient pulse in the heat flux signal corresponds to an energy E = *A*_2·_*τ^2^*. In the exercise shown in Figure 9B, this energy is negative (−43.0 J), while in the post-exercise phase, it is positive (62.2 J). It is important to note that this area is directly proportional to the square of the time constant *τ*. In a simple exponential model, the heat capacity and the time constant are directly related through the skin’s thermal resistance. Therefore, the transient contact energy is closely linked to the thermal dynamics of the heat-affected zone. Since skin heat capacity depends on its composition, and blood perfusion can alter the tissue’s water content (with water having a high specific heat capacity), this transient energy is of great interest. Then, this transient is probably related to changes in muscle blood perfusion at the onset of exercise, leading to a heat flux reduction [41,42,43,44]. In contrast, when exercise ends, hyperemia occurs [8], which explains the heat flux increase at the onset of the recovery phase.

The heart rate is also shown in Figure 8. It was fitted with an exponential function, with a time constant of 12 min during the exercise phase, *bpm* = 83.2 + 80.8 (1 − *e* ^−*t*/12^), and of 5 min post-exercise, *bpm* = 158.6 − 56.8 (1 − *e* ^−*t*/4.6^). Heart rate begins to rise immediately at the onset of exercise; however, there is a temporal delay in the onset of its decline once exercise stops. This delay probably affects the amplitude of the transient pulse during the recovery phase, since thermal conditions may affect the heart rate evolution [45].

### 3.3. Transfer Function and Thermal Simulations

Since the heat flux can be fitted in all cases to a functional expression of the type shown in Equation (6), we can propose a Transfer Function (TF) whose input is the mechanical work performed and whose output is the variation in the local heat flux transmitted through the skin of the thigh. Considering that the time-domain expression in Equation (6) corresponds to the response to a step input of amplitude *W*, the Laplace-domain representation of this TF takes the following form:

(7) $$\begin{array}{l} \mathrm{Input}\;W(s)=\int_{0}^{\infty }W\cdot e^{-st}dt=\frac{W}{s}\\ \mathrm{Output}\;\Delta W_{1}(s)=\int_{0}^{\infty }\left[A_{1}\left(1-e^{-t/\tau }\right)+A_{2}te^{-t/\tau }\right]e^{-st}dt=\frac{A_{1}}{s\left(1+s\tau \right)}+\frac{A_{2}\tau^{2}}{{\left(1+s\tau \right)}^{2}}\\ TF(s)=\frac{Output}{Input}=\frac{\Delta W_{1}(s)}{W(s)}=\frac{1}{W}\left[\frac{A_{1}}{1+s\tau }+\frac{A_{2}\tau^{2}s}{{\left(1+s\tau \right)}^{2}}\right]=K\frac{1+s\tau *}{{\left(1+s\tau \right)}^{2}} \end{array}$$

In this expression, the coefficients *A_1_* and *A_2_* and the time constant *τ* are the parameters determined and listed in Table 2 for a mechanical power output of W = 80 W. With this functional form (Equation (7)), the skin heat flux *W_1_*(t) can be determined for any exercise protocol *W*(t) performed by the subject. As an example, we present an application intended to illustrate the potential usefulness of this Transfer Function, noting that it is valid only for the subject studied. First, the Transfer Function is defined in MATLAB. The results of this study (Table 2) lead us to propose an average Transfer Function for the subject, valid across the entire range of thermostat temperatures used (Equation (8)).

(8) $$\begin{array}{ll} \mathrm{Exercise} & TF(s)=0.9773\frac{\left(1-338s\right)}{{\left(1+365.8s\right)}^{2}}\left(\frac{mW}{W}\right)\\ \mathrm{Post-recovery} & TF(s)=-0.6244\frac{\left(1-551s\right)}{{\left(1+572.3s\right)}^{2}}\left(\frac{mW}{W}\right) \end{array}$$

**Figure 10:**
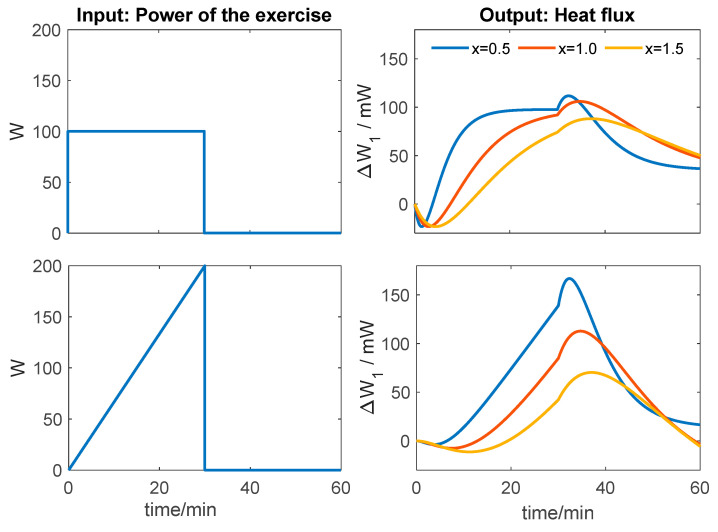
Simulated increases in thigh heat flux during exercise and post-exercise recovery. Step input and ramp input cases for the Transfer Functions are described by Equation (9).

Then, we generate the input function (exercise protocol), which can be a step, a ramp, or any arbitrary shape. Finally, using MATLAB’s *lsim* function, the corresponding response (skin heat flux) is obtained. Figure 10 shows the simulation results for two scenarios: constant power exercise and ramp power exercise. Three cases were simulated with the same sensitivity: nominal TFs (for x = 1), TFs with halved time constants (x = 0.5), and TFs with time constants 1.5 times larger (x = 1.5).

(9) $$\begin{array}{ll} \mathrm{Exercise} & \mathrm{Post}-\mathrm{recovery}\\ TF(s)=0.977\frac{\left(1-338xs\right)}{{\left(1+365.8xs\right)}^{2}} & TF(s)=-0.624\frac{\left(1-551xs\right)}{{\left(1+572.3xs\right)}^{2}} \end{array}$$

It can be observed that the responses to a step input are similar in shape but exhibit clearly different dynamics that can be directly evaluated. In contrast, responses to ramp inputs are harder to interpret directly and require more sophisticated mathematical treatment to analyze the dynamics and determine the corresponding Transfer Function. Currently, progressive ramp exercise protocols are commonly used to determine the ventilatory threshold and assess cardiorespiratory efficiency [46]. However, to characterize muscle intrinsic dynamics, a step input with moderate power may be more appropriate.

### 3.4. Discussion

The skin calorimeter addresses the main limitations of commercial heat-flux sensors (HFSs) in measuring skin heat flux, by minimizing disturbances from convection, radiation, and air currents. In contrast to HFSs, the skin calorimeter incorporates a controlled reference, the thermostat, which provides a stable baseline and improves measurement accuracy. Table 4 compares the technical specifications of the skin calorimeter with commercial HFSs. The skin calorimeter shows a higher sensitivity (195 mV/W) than thin-film sensors, and it is also higher than plate-type sensors. Its 4 cm^2^ measurement area offers a good balance: it is large enough to provide a stable and strong signal but small enough to keep local specificity without losing detail. In addition to direct heat flux measurement, the calorimeter can estimate skin heat capacity, thermal resistance, and subcutaneous temperature. Although the skin calorimeter requires specialized instrumentation and is more complex to operate, the prototype cost is similar to standard HFSs.

This work describes the calorimeter and its operation and introduces a new skin calorimetry application: the measurement and modeling of localized muscle heat flux during exercise. The proposed model (Equation (6)) is consistent with approaches based on the Pennes bioheat equation [24,25,26,27,28,29,30,31,32,33,34,35,36]. The incorporation of a linear–exponential term allows for the identification of the transients at the onset and end of exercise. Although linear–exponential terms are not typically used in human thermal modeling, similar functions (second-order gamma variate forms) have been applied in other fields—for instance, to describe acoustic intensity echography responses to induced vascular perturbations [50,51] or to characterize neural mass models describing the postsynaptic response to an impulse [52,53]. 

On the other hand, using TFs allowed us to identify the experimental responses and simulate exercise scenarios (Figure 10). This predictive capability is potentially valuable for clinical applications, as it characterizes the subject’s intrinsic thermal dynamics, although it remains limited to the identified individual.

One limitation of this study is that only one subject was evaluated, which restricts the generalizability of the findings. While this design allowed for control over intra-individual variability, further research with a broader sample is needed to validate the model and confirm its clinical applicability. Inter-individual differences in subcutaneous fat, tissue composition, and blood perfusion may influence skin thermal resistance, time constants, and the transient energy term, thereby affecting both the magnitude and dynamics of the measured heat flux. A second important limitation is sweating, since the superposition of this phenomenon (evaporation) produces a decrease in the measured heat flux (by conduction). To avoid this, exercise intensity was limited to moderate levels. For this reason, the environmental conditions must also be controlled.

Despite these limitations, and considering the complexity involved in conducting extensive human studies, we believe it is appropriate to publish the model at this stage, as it may serve as a useful starting point for future simulations and experimental validation.

## 4. Conclusions

This case study demonstrates the feasibility of using a skin calorimeter to measure localized muscle heat flux—and, indirectly, subcutaneous temperature—during exercise, and to model its temporal evolution. The proposed two-exponential model, which includes a novel linear–exponential term, successfully describes both heating and recovery phases, as well as the transient phenomena. From the fitted parameters, a subject-specific Transfer Function was obtained, allowing for the simulation of heat flux responses to different exercise profiles. These results confirm the method’s potential for controlled experimental studies and future applications in sports and clinical contexts. 

## Figures and Tables

**Table 1:** Calorimetric model (Equation (1)) and cooling system (Equation (2)) parameters.

Parameters		Calorimeter *S1*		Calorimeter *S2*		Units
		Mean	±std	Mean	±std	
**RC model**	***C_10_***	2.31	±0.07	2.31	±0.07	J/K
*C_1_*		4.02	±0.09	3.91	±0.09	J/K
*C_2_*		3.8	±0.2	3.7	±0.3	J/K
*P_1_*		0.029	±0.002	0.029	±0.002	W/K
*P_2_*		0.057	±0.005	0.055	±0.005	W/K
*P_12_*		0.092	±0.008	0.089	±0.009	W/K
*k*		23.7	±1.1	23.0	±1.4	mV/K
Cooling system	*α*	13.6		17.4		°C/A
*β*		−83.5		−83.8		°C/A
RMSE values	*ε* *_y_*	16.5	±2.5	16.2	±2.4	µV
*ε* *_T2_*		3.9	±2.0	3.8	±2.0	mK

Note: 35 calibration measurements were performed, each one with 2700 data points (45 min).

**Table 2:** Fitting of heat flux *W_1_* using Equation (6). Measurement on the thigh (rectus femoris) of a subject performing exercise (80 W) on a stepper, for different thermostat temperatures.

	Exercise					Recovery phase					
*T_2_* °C	*A_0_* mW	*A_1_* mW	*A_2_* mW/min	*τ* min		*A_0_* mW	*A_1_* mW	*A_2_* mW/min	*τ* min		*RMSE* μW
28	125.2	63.7	−21.0	5.7		185.6	−57.20	11.5	9.3		85.4
30	143.4	111.5	−34.3	5.3		251.7	−108.7	−0.02	11.6		78.3
32	86.10	103.0	−41.1	5.3		185.2	−72.19	8.90	8.7		67.3
34	35.40	85.7	−26.5	5.2		118.7	−48.35	20.0	7.2		102.9
36	61.50	75.4	−33.5	5.6		132.9	−42.91	4.40	12.0		83.6
38	−24.53	29.8	−17.0	6.7		0.200	29.66	21.7	6.3		105.7

**Table 3:** Fitting of subcutaneous temperature *T_core_* using Equation (6). Measurement on the thigh (rectus femoris) of a subject performing exercise (80 W) on a stepper, for different thermostat temperatures.

	Exercise					Recovery phase					
*T_2_* °C	*A_0_* °C	*A_1_* °C	*A_2_* °C/min	*τ* min		*A_0_* °C	*A_1_* °C	*A_2_* °C/min	*τ* min		*RMSE* mK
28	33.15	2.61	−0.99	5.7		35.61	−2.27	0.56	9.2		3.56
30	35.00	4.45	−1.53	5.4		39.30	−4.39	0.00	13.8		3.01
32	33.75	3.96	−1.63	5.4		37.54	−2.58	0.42	8.5		2.54
34	33.52	3.05	−1.06	5.2		36.48	−1.62	0.79	7.3		3.84
36	35.71	2.46	−1.23	5.7		38.01	−1.30	0.22	10.7		2.73
38	34.05	0.82	−0.56	6.7		34.71	1.25	0.72	6.5		3.35

**Table 4:** Technical specifications of different heat-flux sensors.

	Measurement (cm^2^)	Thickness (mm)	Sensitivity (mV/W)
Film Heat flux HFS-4 [47]	10.0	0.18	2.1
Film Heat flux HFS-5 [48]	6.3	0.36	2.2
Film Heat flux FHF05 [49]	1.0	0.40	10.0
Film Heat flux FHF05 [49]	4.5	0.40	6.6
Heat flux plate HFP01 [49]	8.0	5.40	75.0
Skin Calorimeter (this work)	4.0	2.20	195

## Data Availability

All data underlying the results are available as part of the article, and no additional source data are required.

## Abbreviations

The following abbreviations are used in this manuscript:

PID control	Proportional–Integral–Derivative Controller
RMSE	Root Mean Square Error
GPIB	General Purpose Interface Bus
TF	Transfer Function
HFS	Heat-Flux Sensor
IRT	Infrared Thermography
NIRS	Near-Infrared Spectroscopy
EPS	Expanded Polystyrene
bpm	Beats Per Minute (Heart Rate)
